# Weather and agricultural intensification determine the breeding performance of a small generalist predator

**DOI:** 10.1038/s41598-020-76609-x

**Published:** 2020-11-12

**Authors:** Paula M. Orozco-Valor, Juan M. Grande

**Affiliations:** 1 Instituto de las Ciencias de la Tierra y Ambientales de La Pampa (INCITAP)-Consejo Nacional de Investigaciones Científicas y Técnicas de Argentina (CONICET), Mendoza 109, 6300 Santa Rosa, La Pampa Argentina; 2 Centro para el Estudio y Conservación de las Aves Rapaces en Argentina (CECARA), Facultad de Ciencias Exactas y Naturales, Universidad Nacional de La Pampa, Avenida Uruguay 151, 6300 Santa Rosa, La Pampa Argentina; 3 Facultad de Ciencias Exactas y Naturales, Universidad Nacional deLa Pampa, Avenida Uruguay 151, 6300 Santa Rosa, La Pampa Argentina

**Keywords:** Ecology, Zoology, Climate sciences, Ecology, Environmental sciences

## Abstract

Land-use changes due to agricultural intensification and climatic factors can affect avian reproduction. We use a top predator of agroecosystems, the American kestrel (*Falco sparverius*) breeding in nest boxes in Central Argentina as a study subject to identify if these two drivers interact to affect birds breeding. We analyzed their breeding performance across a gradient of agricultural intensification from native forest, traditional farmland to intensive farmland. The surface devoted to soybean was used as a proxy of agriculture intensification; however, it did not affect the breeding performance of American kestrels. Even though the presence of pastures was important to determine the probability of breeding successfully. Climatic variables had strong effects on the species breeding timing, on the number of nestlings raised by breeding pairs and on the probability of those pairs to breed successfully (raising at least one fledgling). Our results highlight the relevance of pastures and grasslands for American kestrel reproduction. These environments are the most affected by land-use change to intensive agriculture, being transformed into fully agricultural lands mostly devoted to soybean production. Therefore, future expansion of intensive agriculture may negatively affect the average reproductive parameters of American Kestrels, at least at a regional scale. Further research will be needed to disentangle the mechanisms by which weather variables affect kestrel breeding parameters.

## Introduction

Understanding why some animal populations thrive while others decline or disappear is a central topic in ecology. For this reason, it’s necessary to study the demographic parameters of animal populations and the factors that shape them as they determine the abundance and distribution of those populations^1,2^. Reproduction is one of the central demographic parameters since it determines the passage of genes from one generation to the next^3^.

One of the main causes of animal population declines is habitat destruction and degradation which alters the original ecosystems and thus the relationships of those animals with their environment^4^. Agriculture has globally become the dominant land use, transforming the world’s surface at an accelerated rate^5,6^. The substitution of native vegetation cover by crops imposes changes in all the ecological relationships within the altered ecosystems. Besides the effects of the drastic land cover change, in recent decades, intensification processes in farming practices (extreme mechanization, genetically modified crops and massive use of agrochemicals) have also contributed to a reduction in the surface devoted to extensive livestock farming and of traditional livestock/crop rotation systems and their conversion to exclusive and intensively farmed cropland. Modern agricultural practices allowed this transformation even in traditionally marginal and unproductive areas thus expanding its impacts widely and into areas previously spared from agriculture^7^.

Given their naturally low abundances, large home ranges, and their position as top predators that may facilitate bioaccumulation processes, birds of prey may be particularly sensitive to major ecosystem changes such as those produced by agricultural expansion and intensification^8,9^. The effects of this industrial agricultural production on raptor populations are diverse and may affect their abundance patterns^10^, destroy potential nesting or foraging habitats^11^, affect nestling conditions^12^, cause direct mortality by pesticides or other farming practices^13,14^ and reduce breeding performance^15,16^. But at the same time, it seems that agroecosystems can still provide suitable habitat for some raptors associated with increases in the availability of particular food items and breeding resources^17,18^. For example, the Varreaux eagle (*Aquila verreauxii*) is a highly specialized predator of hyraxes, a group of small mammal species usually associated to rock outcrops and bushes and absent from agricultural lands. However, in agricultural lands the eagle diversified its diet with a positive effect on breeding performance^18^. Another example is the Western marsh harrier (*Circus aeruginosus*), a ground nesting raptor that use human constructed irrigation ponds for breeding in agricultural regions and exploit surrounding irrigated crops as hunting habitats where they find abundant rodents^17^.

Besides land cover changes introduced by humans, abiotic factors such as climate also have critical implications on the regulation of wild bird populations^19,20^. Rainfall, for example, could determine food availability through boosting primary production or by shaping raptor foraging or prey activity^21,22^. The impacts of rainfall on breeding raptors, depends on its intensity and may be different across habitats. Lower levels of rainfall in a Mediterranean landscape, negatively affected breeding success of Hen harriers (*Circus cyaneus*) probably through mediated food resources^23^. In tropical environment, increases of precipitations in spring delayed the onset of breeding of some species like the Mauritius kestrel (*Falco punctatus*) and thus, adversely affected breeding success^24^. Temperatures is also a critical environmental variable that has strong impacts in bird’s reproduction determining in many cases the bird´s laying date^25,26^. Temperature conditions experienced during the breeding cycle could affect raptors breeding performance in several ways^27^, including nest occupation^28^, egg laying^29^, or breeding success^30^. Lower temperatures experienced during winter combined with lower food abundance delay egg laying in Golden eagle (*Aquila chrysaetos*) in semiarid climates in western North America^29^, while warmer winter and early spring temperatures in Mediterranean environments favored Northern goshawk (*Accipiter gentilis*) productivity^30^. Cold temperatures increased chick mortality, female brooding and combined with high levels of rainfall negatively affected male provisioning rate in Hen harries in Scotland^31^.

In the last three decades, several countries in Latin America promoted a strong agriculture expansion and intensification process. In Argentina, large extensions of dry forests in the Chaco and the Espinal as well as of natural grasslands have been turned into crops, mainly soybean^32,33^. However, it is not clear how the emergence of these intensive production systems or their interaction with climate could affect bird species and particularly top predators such as raptors. Studies on Argentinean bird of prey communities indicate they are less diverse and abundant in areas of agricultural production than in grassland areas^34–36^. However, few studies have addressed in this country habitat selection by raptors at a specific level and as far as we know none has assessed the combined effects of weather and land cover variables on raptor’s breeding biology.

The American kestrel (*Falco sparverius*) is one of the commonest birds of prey all across its breeding range and can be found in a wide variety of habitats from northern Canada to Tierra del Fuego, including heavily populated cities^37^. Recent evidence indicate that their numbers are declining across several areas of North America^38^. Although the reasons are still unclear, the effects of agricultural intensification are among the studied candidate factors^16,38,39^. In Argentina, the species is considered resident and occurs throughout the country, being especially abundant in agroecosystems and other open areas^40^. Even though traditionally linked to agroecosystems, in this country some studies suggest that American kestrels prefers grassland and avoids or is less common on farmland^34^, which would suggest the species could be adversely affected by agricultural expansion and intensification processes. Other studies suggest that it is more abundant in farmland^41,42^ and even favored in areas of soybean production^43^.

In this context, the current study aimed to assess possible effects of intensified agriculture and weather (rainfall and temperature) in free-living American kestrels breeding in nest boxes in central Argentina. We compared American kestrels nest-box occupancy and breeding performance in a gradient of land-use intensification across three sampling areas, a native forest (Parque Luro Natural Reserve-PLNR), a traditional farmland (TF) and an intensive farmland (IF, Fig. 4).

Given that kestrels prefer open spaces for hunting we expected that nest box occupancy would be higher in agricultural lands. As the landscape is more homogeneous and receives a higher load of agrochemicals, we expected occupation and breeding parameters in IF (areas with a high percentage of soybean and less cover of pasture or other crops) to be lower than TF (areas with a lower percentage of soybean and higher percentage of pastures and other crops) or in PLNR (an area covered by native forest). Given that temperature and rainfall can also affect breeding parameters, we also analyzed their effects on laying date, clutch size, productivity (number of fledglings raised per breeding pair) and breeding success (probability of raising successfully at least one fledgling). We considered the effects of rainfall and temperature during the pre-laying period (winter) and the laying period (spring) on laying date and clutch size. We expected that higher rainfall and lower temperatures during the egg laying period would negatively affect adults foraging activity or prey availability affecting females body condition and thus delaying the onset of laying and constraining the clutch size of kestrels. To account for the effect of weather on productivity and breeding success we considered the rainfall and temperatures registered during the nestling period (late spring and early summer). High rainfall and temperatures during the nestling period may induce physiological stress to the nestlings and reduce adults foraging possibilities, therefore we predicted that higher temperatures and rainfall during the nestling period would negatively affect American kestrels’ productivity and the probability of breeding pairs raising at least one fledgling successfully. Furthermore, as most chicks hatch in November, we expected that high rainfall and temperatures in this month could be particularly negative for productivity and for the probability of breeding successfully. If the preceding winter severity negatively affects egg laying and clutch size, then the rainfall and temperature during the winter should also influence negatively the number of nestlings produced and the probability of breeding successfully. Since the onset of reproduction is important to determine breeding success, we expected that low temperatures during the laying period will also have a negative effect on breeding success.

## Results

### Land uses

**Figure 1:**
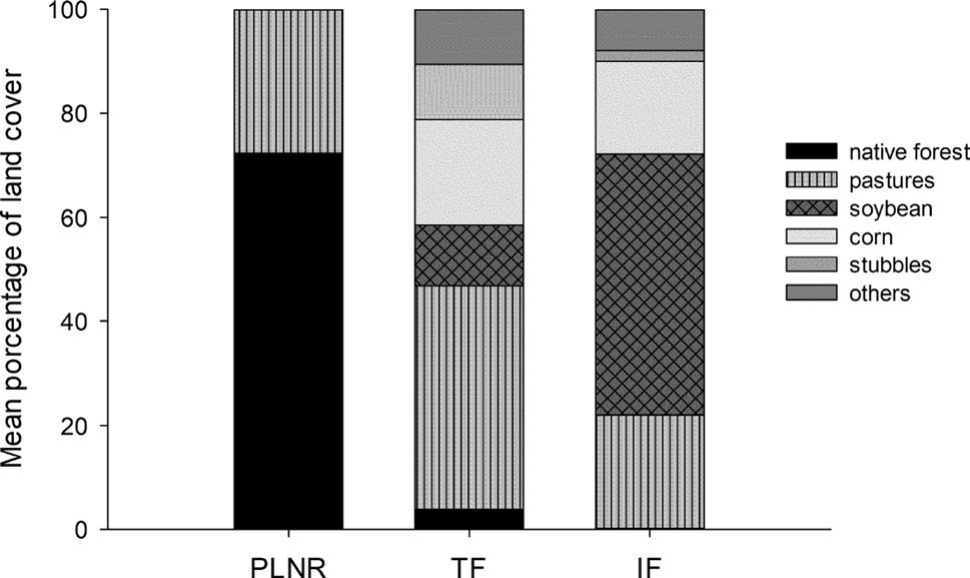
Mean percentage of the different land uses present in the 500 m radius buffers surrounding each nest box in the three sampling areas during 2012 and 2014–2016 in La Pampa (PLNR = Parque Luro Natural Reserve, TF = Traditional farmland, IF = Intensive farmland).

Soybean covered on average half of the surface of 500 m buffers (territories assumed to have a 500 m radius following references, see methods) around nest boxes in the intensive farmland area (IF) through the breeding seasons (2012 and 2014–2016) (Fig. 1), although the surface ranged from 100% soybean cover in some nest boxes to less than 1% in others. Pastures were the second land use in abundance. In the traditional farming area (TF) only 10–15% of the surface surrounding nest boxes had soybean, and pastures dominated the landscape. In both farming areas, the second crop in surface cover was corn and, the remaining surface was covered by other crop types. In PLNR, Caldén (*Prosopis caldenia*) forest covered 70% of the surface around nest boxes followed by natural pastures (Fig. 1).

During the pre-laying stage (June to August), the mean minimum temperature in our study area was 4.28 ± 0.21 °C (± SE) and the mean precipitation was 60.03 ± 8.51 mm (± SE). During the egg laying period (Mid-September and October), the average temperature recorded was 14.84 ± 0.43 °C (± SE) and mean precipitation was 217.89 ± 14.10 mm (± SE). During the chick rearing period (November and December) the mean temperature recorded was 21.97 ± 0.39 °C (± SE) and the mean precipitation was 151.85 ± 20.02 mm (± SE).

### Breeding

From 2011 to 2016 we monitored a total of 457 American kestrel breeding attempts in central Argentina. Although a few breeding pairs laid eggs in mid-September and a couple of late breeder’s fledglings left the nests in mid-February most pairs bred from mid-October to mid-January (Table 1).

**Table 1:** Summary of reproductive parameters of American kestrels in central Argentina per study site. General data and data from each sampling areas (PLNR = Parque Luro Natural Reserve, TF = Traditional farmland and IF = Intensive farmland) is provided. Mean values, the standard error (± ES) and the number of nest boxes monitored (in brackets) from 2011 to 2016 are provided.

Sampling site	Laying date	Clutch size	Productivity	Breeding success
Parque Luro Natural Reserve	21 October ± 1.96 (n = 36)	4.16 ± 0.17 (n = 48)	2.62 ± 0.31 (n = 45)	0.68 ± 0.06 (n = 45)
Traditional farmland	18 October ± 0.84 (n = 239)	4.63 ± 0.04 (n = 257)	3.33 ± 0.1(n = 256)	0.86 ± 0.02 (n = 256)
Intensive farmland	18 October ± 1.32 (n = 106)	4.29 ± 0.06 (n = 141)	2.57 ± 0.16 (n = 140)	0.69 ± 0.03 (n = 140)
General	18 October ± 0.67 (n = 381)	4.48 ± 0.04 (n = 446)	3.02 ± 0.09(n = 441)	0.79 ± 0.02 (n = 441)

**Figure 2:**
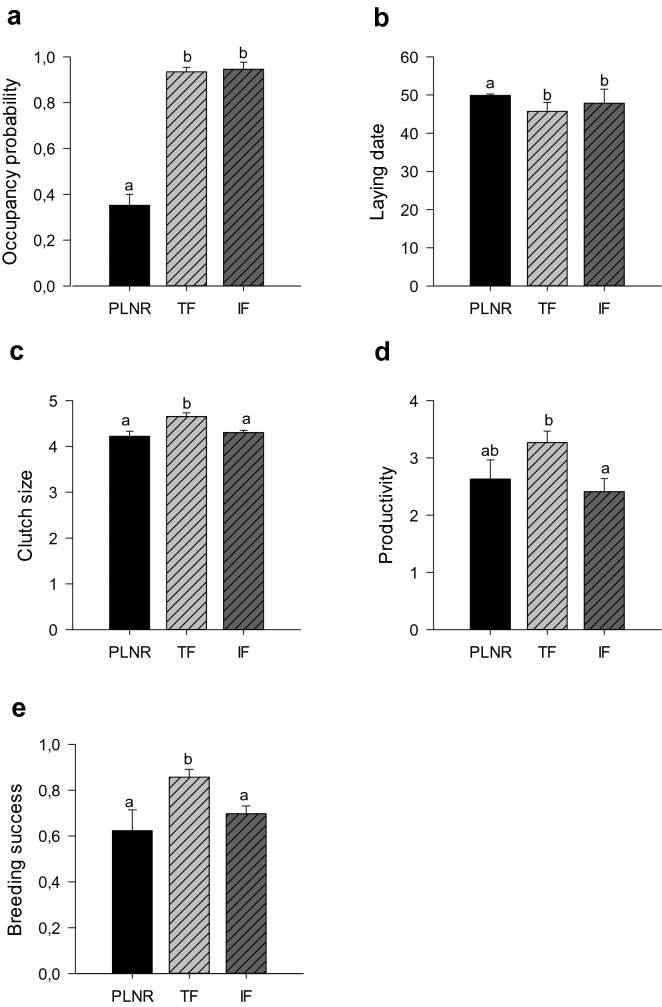
Observed values (± ES) of nest box occupancy (**a**), laying date (**b**), clutch size (**c**), productivity (**d**) and breeding success (**e**) of American kestrels between sampling areas. (PLNR = Parque Luro Natural Reserve, TF = Traditional farmland, IF = Intensive farmland ). Significant differences (p < 0.05) as a result of the Tukey test are indicated with different letters above the bars or plots. Day 0 in laying date correspond to September the 1st, and thus mean laying dates in the graph are between October the 10th (day 40) and October the 20th (day 50).

Every year, since 2011 a high percentage of nest boxes were occupied by American kestrels in both agricultural lands while the percentage of occupancy in PLNR was lower (Table 2). Nest box occupation was determined by the sampling areas (X^2^ = 30.86, df = 2, p < 0.001, Fig. 2a). Post hoc comparisons indicated that the occupation rate was similar between both agricultural areas (z = − 0.46, p = 0.795) and higher there than in PLNR (z = 5.33, p < 0.001 and z = 5.03, p < 0.001, TF and IF, respectively).

**Table 2:** Nest box occupancy by American kestrels among sampling areas (Parque Luro Natural Reserve, Traditional farmland and, Intensive farmland) between 2011 and 2016 in La Pampa, central Argentina. Nest boxes in intensive agricultural areas were placed in the year 2012. The number of nest boxes provided in each area is indicated in brackets. Given that some nest boxes were stolen or got broken through the different years, we replaced them, however, it generated a slight variation in the final number of nest boxes monitored each year in the different areas.

Year	Parque Luro Natural Reserve	Traditional farmland	Intensive farmland
2011	16.66% (n = 24)	66% (n = 50)	
2012	25% (n = 24)	90% (n = 50)	83.33% (n = 30)
2013	25% (n = 24)	92% (n = 50)	93% (n = 30)
2014	50% (n = 24)	98% (n = 50)	100% (n = 30)
2015	41.67% (n = 24)	100% (n = 50)	100% (n = 31)
2016	45.83% (n = 24)	92% (n = 50)	96.88% (n = 32)

**Figure 3:**
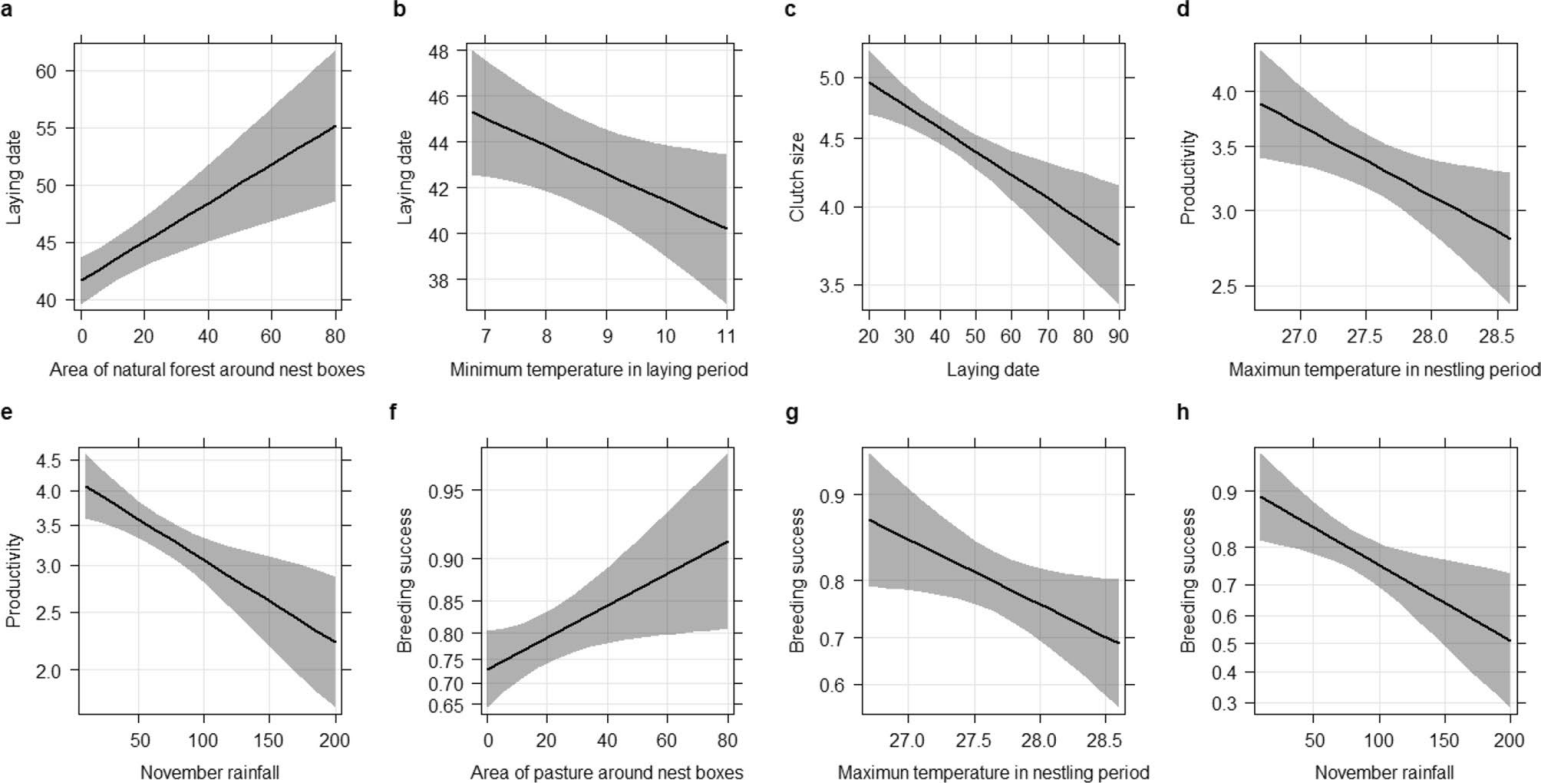
Effects predicted on laying date (**a**,**b**), clutch size (**c**), productivity (**d**,**e**) and on breeding success (**f**–**h**) of the different variables selected in the GLMM and LMM used to evaluate variation in American kestrels breeding parameters in relation to weather and land use during breeding season 2012 and 2014–2016 in La Pampa, central Argentina. Unconditional confidence intervals 95% around predictions are shown in grey. Day zero in laying date correspond to September 1st.

American kestrels in central Argentina initiated egg-laying on average (± SE) on 18 October ± 0.67 days (Tables 1 and 3). Laying date varied among sampling areas (X^2^ = 11.09, df = 2, p = 0.038, Fig. 2b) being later in PLNR than in both agricultural lands (z = − 3.31, p = 0.002 in TF and z = − 2.43, p = 0.030 in IF) but without differences between agricultural lands (z = 1.09, p = 0.511). The inclusion of the different land uses in the models indicated that higher cover of native Caldén forest was associated with delayed laying date (X^2^ = 14.50, df = 1, p < 0.001, Fig. 3a). Colder minimum temperatures registered during the laying period (Tmin_laying) was also associated with later laying dates (X^2^ = 4.66, df = 1, p = 0.031, Table 4 and Supplementary Table S1 online, Fig. 3b).

**Table 3:** Summary of general reproductive parameters of American kestrel per year from 2011 to 2016. The mean values, the standard error (± ES) and the number of nest boxes in brackets are provided. Nest boxes in the intensive agricultural area were placed in 2012.

Year	Laying date	Clutch size	Productivity	Breeding success
2011	31 Oct + 1.91 (n = 24)	4.22 + 0.20 (n = 35)	3.53 + 0.28 (n = 31)	0.87 + 0.06 (n = 31)
2012	26 Oct + 1.4 (n = 64)	4.30 + 0.1 (n = 75)	2.65 + 0.23 (n = 71)	0.70 + 0.05 (n = 71)
2103	21 Oct + 1.06 (n = 51)	4.54 + 0.08 (n = 78)	2.98 + 0.22 (n = 78)	0.87 + 0.06 (n = 78)
2104	11 Oct + 1.54 (n = 81)	4.69 + 0.10 (n = 89)	3.39 + 0.18 (n = 89)	0.86 + 0.03 (n = 89)
2015	14 Oct + 1.30 (n = 87)	4.46 + 0.07 (n = 90)	3.27 + 0.18 (n = 86)	0.83 + 0.04 (n = 86)
2106	17 Oct + 1.50 (n = 68)	4.44 + 0.09 (n = 81)	2.55 + 0.21 (n = 85)	0.72 + 0.04 (n = 84)

**Table 4:** Result from GLMM and LMM to evaluate variation in breeding performance (laying date, clutch size, productivity, and breeding success) of American kestrels in relation to rainfall, temperature and the different land uses in the buffer of 500 m around nest boxes in the center of Argentina from 2012, and 2014 to 2016.

Models	Estimate	± ES	X^2^	df	P value
**a. Laying date**					
Intercept	52.071	4.963			
Forest	0.169	0.045	14.508	1	< 0.001
Tmin_laying	− 1.211	0.565	4.666	1	0.031
**b. Clutch size**					
Intercept	1.168	0.048			
Laying date	− 0.004	0.001	14.065	1	< 0.001
**c. Productivity**					
Intercept	6.156	1.958			
November rain	− 0.003	0.001	11.342	1	< 0.001
Tmax_nestling	− 0.171	0.069	6.014	1	0.014
**d. Breeding success**					
Intercept	18.762	8.291			
Pasture	0.017	0.007	5.056	1	0.024
November rain	− 0.010	0.003	7.436	1	0.006
Tmax_nestling	− 0.061	0.029	4.376	1	0.036

The mean clutch size (± SE) for American kestrels in our study area was 4.48 ± 0.04 (Tables 1 and 3). Clutch size varied among sampling areas (X^2^ = 15.34, df = 2, p < 0.001, Fig. 2c). Post hoc comparisons indicated that clutch size varied among sampling areas, being larger in TF than in IF (t = 3.22, p = 0.039, Fig. 2c) and PLNR (t = − 2.93, p = 0.009) but without differences between PLNR and IF (t = − 0.77, p = 0.717). Clutch size was negatively related with laying date so that early breeders had larger clutches than late breeding pairs (X^2^ = 14.06, df = 1 p < 0.001, Table 4, Fig. 3c). We did not find an effect of rainfall; temperature or land uses on clutch size (p > 0.05 in all cases, Supplementary Table S1 online).

From a total of 394 clutches analyzed, 19.79% present total hatching failure (all eggs in a clutch did not hatch) and 36.04% presented some hatching failure (some eggs in a clutch did not hatch) while only 10.12% of 316 broods suffered brood reduction.

Productivity, measured as the number of fledglings that fledge per pair attempting breeding, was on average (± SE) 3.02 ± 0.1 nestlings per occupied nest box (Tables 1 and 3). Productivity varied between sampling areas (X^2^ = 14.5, df = 2, p < 0.001, Fig. 2d). Particularly, productivity varied between both agricultural lands (Fig. 2d), being the number of nestlings in TF higher than in IF (t = 3.5678, p = 0.001), while no differences were found between PLNR and both agricultural lands (t = − 2.67, p = 0.09 in TF and t = 0.14, p = 0.98 in IF). Productivity was negatively associated with mean maximum temperatures registered during the nestling period (X^2^ = 60.01, df = 1, p = 0.014, Table 2, Fig. 3d) and with rainfall in November (X^2^ = 11.34, df = 1, p < 0.001, Table 2, Fig. 3e). We found a marginally non-significant negative effect of laying date on productivity (X^2^ = 3.606, df = 1, p = 0.057, Supplementary Table S1 online).

Breeding success, pairs that successfully reared at least one nestling from the pairs that started breeding, was on average (± SE) 0.79 ± 0.02 (Tables 1 and 3) and varied between sampling areas (X^2^ = 18.91, df = 2, p < 0.001, Fig. 2e). We found higher breeding success in TF than in PLNR (z = 3.20, p = 0.003, Fig. 2e) and IF (z  = − 3.95, p < 0.001, Fig. 2e), while there were no differences between IF and PLNR (z = 0.50, p = 0.865). Breeding success was positively associated with the surface of pastures around nest boxes (X^2^ = 5.056, df = 1, p = 0.024, Table 4 and Supplementary Table S1 online, Fig. 3f) and negatively associated with the mean maximum temperatures during the nestling period (X^2^ = 4.376, df = 1, p = 0.036, Table 4, Fig. 3g) and rainfall in November (X^2^ = 7.43, df = 1, p = 0.006, Table 4, Fig. 3h). Therefore, pairs with more pastures around the nest box had more probability of breeding successfully, as did pairs in years with lower maximum temperatures during the breeding season and with lower rainfall in November.

## Discussion

Variation in weather and land uses generated differences in the breeding performance of a generalist predator through a gradient of agriculture intensification in central Argentina. Temperature conditions throughout the laying and chick rearing period affected the American kestrel breeding timing, the number of fledglings produced per nest and the probability of raising successfully at least one fledgling, while precipitation during November had a negative effect on their probability of breeding successfully. Besides a high occupation rate of nest boxes in agricultural lands, our findings indicate that the presence of pastures and grasslands in breeding territories had a positive effect on the probability of breeding successfully. We found that both breeding success and productivity were higher in the traditional farmland where pastures and grasslands are the dominant cover, than in the intensive farmland (where soybean is prevalent), with intermediate values in the more closed environments of the forest area. Given that the conversion of pastures into intensive crop farming is the main land-use change in the Argentinean former grasslands^44^, our results indicate that intensive farming is affecting American kestrels breeding output through the reduction in pastures cover, but apparently not by the type of crop.

American kestrel breeding performance in central Argentina was similar to previous reports for the species in the country in native forests in our study area^45^ and in Patagonia^46^. However, in our study, taking into account early and late breeders, breeding extended from mid-September to mid-February, indicating a much longer reproductive season than reported in previous studies. These differences may be due to the small sample size analyzed in previous studies since both, Liébana et al.^45^ and De Lucca and Saggesse^46^ only monitored sixs nest during a single breeding season, contrasting with the 457 reproductive events monitored over 6 breeding seasons in the present study. On the other hand, the results of this study also coincided with the general reproductive parameters reported for the American kestrels in the northern hemisphere^16,47^.

Nest box occupation by American kestrels in both agricultural farmlands (traditional and intensive) was greater than in PLNR. American kestrels prefer nests located in open habitats^47^ and some nest boxes in PLNR were put up in areas of closed forest. In fact, nest box occupancy by raptors in PLNR, mostly American kestrels, was positively related with the cover of open pastures in their surroundings^48^. Also, the high percentage of nest box occupancy over 80% (and over 90% from the second year of nest boxes presence ahead) in both agricultural lands may be attributed to the lack of appropriated natural cavities in this landscape. The low occupancy of nest boxes in Caldén forest (from around 17% to 50%) could also reflect the high availability of alternative breeding sites there. The American kestrel is a secondary cavity nester and thus its populations could be limited by cavity availability^47^. In Parque Luro Natural Reserve, the forest covers more than 70% of the surface (Fig. 1) and thus, kestrels have high availability of alternative nesting sites like tree cavities and nests of Monk parakeets (*Myiopsitta monachus*) and Brown cacholotes (*Pseidoseisura lophotes*), that are also used by the species to breed^49,50^. Nest box occupation in the Pampean agricultural areas was extremely high ranking among the largest reported for the species in its entire range^39,51^. This high rate of nest box occupation in agricultural areas is likely to reflect both the abundance of the species in these environments, given the high occupation recorded since the beginning of the study, and the low availability of adequate cavities in these areas in relation to the enormous availability of open habitats for foraging.

Laying date is generally influenced by food availability^52,53^ that ultimately allow breeding females to achieve the necessary body condition to initiate the clutch. We found that mean laying dates were a few days later in the forest population than in agricultural lands while in the analysis including land cover variables, the cover of the forest had a negative effect on laying dates. The American kestrel is an open space forager and thus, large Caldén forest cover in the territories probably reduce their foraging area as found in other study sites^54^ explaining at least partially this small delay. We did not find a negative effect of heavy rainfall on laying date. However, increases in the minimum average temperatures registered during the laying period were associated with earlier laying dates. Warmer springs have been related with the advance in the initiation of breeding in several avian species^55^. In our study area the temperate-semiarid climate, is characterized by a marked seasonality with well-defined summer and winter seasons^56^. Warmer temperatures in a period where the first spring rains start, may activate primary productivity and the entire food chain on which kestrels depend for laying. However, experimental studies suggest that warmer temperatures can affect directly the onset of laying in birds, probably linked to the activation of determined physiological processes^55^. In the northern hemisphere, American kestrels respond to primary production related to changes in food availability advancing their nesting phenology in areas of irrigated crops, where the growing season has advanced because farmers plant their crops earlier after warmer winters^57^.

Mean clutch size, 4.48 eggs in our study area was similar to those registered in North America populations that are usually between 4–5 eggs, but more close to 4 in southern latitudes (e.g. 4–4.31 in Central Florida) and more close to 5 in northern latitudes (e.g. 4.81 in Canada)^47^. Our results thus are in the middle. However, we registered some cases of particularly large clutches in our study area rarely found in North America^51^. The clutch size was larger in the traditional farming area than in the intensive farming and the Caldén forest area. Clutch size decreased with the advancement of the breeding season and thus late breeders laid fewer eggs. This is a consistent result among birds as they tend to adjust their breeding phenology to the optimal conditions. Therefore, more experienced and better quality birds adjust better their breeding timing, achieving the necessary condition to lay the eggs earlier in the season while lower experienced or those birds in poorer body condition are energetically constrained and thus, breed later and lay smaller clutches^58^. Supporting this, we also found a trend for early breeding pairs to produce a larger number of nestlings.

Unusual weather, particularly during the winter may affect the survival of birds, mammals, and insects, thus reducing food availability for kestrels^59,60^, and affecting their condition in the following breeding season^61^. However, contrary to our prediction, we did not find an effect of the weather in the preceding winter on clutch size.

The cover of pastures around nest boxes was important to determine the reproductive success of American kestrels, suggesting it is a good foraging habitat. Several studies show that changes in agriculture productive systems have been linked to a decrease in food resources for raptors^15,18,62–64^. However, the persistence of implanted pastures and grassland for extensive cattle, as well as the maintenance of grassland in the shoulders of roads and railways, may maintain high local food availability and probably easy access to prey for raptor species like the American kestrel^11,54,65–67^.

The cover of pastures in the traditional farmland dominated the landscape and decreased sharply in the intensive farmland. Land-use change in the former grasslands of the Pampas is focused on the progressive substitution of pastures by crops, mainly soybean^44,68,69^. This, affect many raptor species, particularly those breeding on the ground such as the Long-winged Harrier (*Circus buffoni*) or the Short-eared owl (*Asio flammeus*)^35,70^ and as we found here, probably to those species that prefer to forage in grassland such as the American kestrel^47^. Despite being lower than in traditional farming areas, breeding parameters of American kestrels in the intensive farmland were still good for the species^47^. However, if the tendency of turning pastures to soybean continue, kestrels breeding success in intensive farmland will probably get lower in the future^16^. As we expected, high temperatures during the nestling period and high levels of precipitation in the month when most nestlings hatch, which is the period most energetically demanding^71^, negatively affected productivity and breeding success of kestrels. Heavy rainfall may reduce nestling survival by affecting them directly, e.g. through the loss of heat and the death by hypothermia in the case of strong rainfall during the early days of life. But these effects can also be indirect through reducing prey activity, prey availability, or a combination of both and thus leading to nestling’s starvation^22,28,72^. In the northern hemisphere, the weather also mediated productivity of American kestrels by altering parental provisioning behavior through limiting the accessibility of kestrels to voles (not the vole abundance that was similar through the years). Also, nestlings exposed to inclement weather were smaller, lighter and had lower survival chances^73^. The presence of high temperatures in summer seems to negatively affect productivity and breeding success in our study area. Higher maximum temperatures experienced during the nestling period may affect nestling survival, probably related to chicks suffering from heat stress^74,75^ and dying from hyperthermia^31,76^ or by changes in prey behavior^29^ or foraging activity^77^ that may result in lower prey delivery and death from starvation. We did not find an effect of the preceding winter severity on productivity or breeding success. Also, we did not find negative effects of low temperatures during the laying period on breeding success. Further research on the availability and accessibility of prey in the different weather contexts, as well as on possible kestrel physiological constrains in response to weather variation will be needed to disentangle the mechanisms by which weather affects kestrels breeding parameters.

Weather and land-use conversion alter biodiversity patterns at large and regional scales^19,78^. In light of the continuous expansion of soybean in Argentina, it is expected that agriculture transformation will negatively affect the average reproductive parameters of American kestrels at least at a regional scale. Regardless of the high proportion of soybean surrounding nest boxes, land conversion in our studied intensive farming area is still not as high as in other parts of the country where soybean covers 90–100% of the land surface^43^. In our intensive farming study area, there are still pastures in some fields devoted to feeding horses and cattle, as well as in field margins where remnants of native vegetation can be found. The persistence of these remnants of native vegetation and pastures may be masking the negative effects of agricultural intensification in our study area and be used by American kestrels to successfully occupy those fields. Simple management practices carried out in other agroecosystems such as maintaining untilled field margins and hedgerows seem positive for raptors^10,66^. Similar practices should be encouraged in our study area to increase the kestrel’s breeding performance. Furthermore, particular studies should be carried out to asses, if breeding experience and individual quality are factors that may affect the probability of breeding successfully and therefore the breeding output of American kestrels. Other potential negative effects of this intensive production system such as the effects of agrochemicals on the health of free-living birds should be also evaluated.^12,79^.

## Materials and methods

### Study area

**Figure 4:**
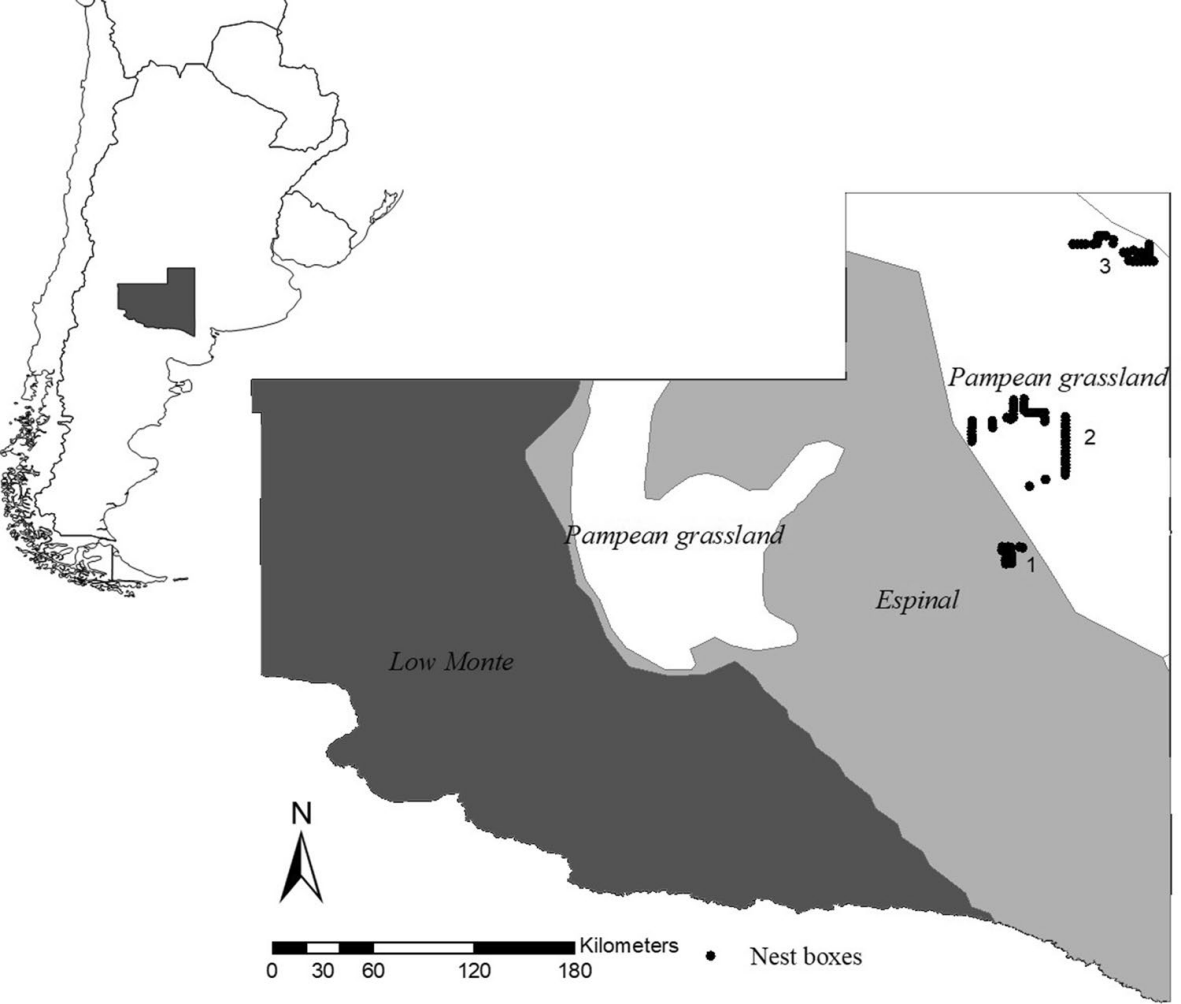
Map of the three sampling areas and nest boxes put up in the north-east of La Pampa province, Argentina (1. PLNR = Parque Luro Natural Reserve, 2. TF = Traditional farmland, 3. IF = Intensive farmland).

The study was carried out in the north-east of La Pampa province of La Pampa province, Argentina (Fig. 4). A total of 104 nest boxes were put up on power line poles along secondary roads at distances of 2 km between each another. The study area covers approximately 14,700 km^2^ of agroecosystems and native semiarid Caldén *(Prosopis caldenia)* forests. In recent decades the area of land devoted to agriculture, and particularly to the growth of soybean increased dramatically. This intensification occurred mostly in the north-east of La Pampa province, where the soil is richer and precipitations higher, thus generating better conditions for agriculture. In the area soybean is the main crop and covers most of the agricultural land, from 38,200 ha 15 years ago, the actual surface devoted to soybean production is around 553,000 ha^80^. To the southwest of the province, rainfall and soil quality decreases and thus intensive farmland is gradually replaced by mixed productive systems (crop and cattle livestock rotation), along with some small isolated patches of Caldén forest. Further south and west, mixed lands become Caldén forests and pastures mainly devoted to livestock production. We worked in three sampling areas (Fig. 4) that differ in landscape composition. Parque Luro Natural Reserve (36°55′ S, 64°16′ W, PLNR, 24 nest boxes) in the center-east of La Pampa, represents a protected area of native Caldén forest. The traditional farming area (36°10′ S, 64°9′ O, TF, 50 nest boxes) formerly covered by open Caldén forest and grassland, is now fully devoted to agricultural production (characterized by the rotation of crops and cattle in a matrix of crops and pastures with little and isolated fragments of Caldén forest^81^). The third area is the intensive farming area (35°16′ S, 63°41′ O, IF, 30 nest boxes) located within the Pampas Grassland ecoregion. The original temperate grasslands^82^ have been almost completely replaced by soybean seeded through intensive agricultural practices such as no-tillage methods. There is also minor presence of other presence of other crops and some remains of seminatural or implanted pastures for cattle, and with the presence of exotic tree stands around settlements. Soybean growing season, from seeding to vegetative growth and flowering, coincides with the American kestrel breeding phenology, beginning the vegetative growth during the nestling period of the kestrels.

The climate is temperate semiarid to subhumid, precipitation increases from center-south-west to north-west, mean annual precipitation ranging from ~ 350 to ~ 850 mm which falls mostly in the spring and autumn, being the native forest sampling area the driest area and the intensive farmland area the wettest^56,83,84^. Mean temperatures range from 15.5 to 16.5 °C^56,84^.

### Habitat analysis and weather data

To analyze the effects of agricultural intensification on the breeding performance of American kestrels, we measured the surface devoted to different land uses in the three sampling areas during four breeding seasons (2012, 2014–2016). Although there is no proper assessment of American kestrel territory size and certainly its size may vary locally depending on habitat quality and nesting site availability, estimates based on distances between occupied nesting sites indicate that ranges of 0.24–0.81 km^2^^85,86^ and diameters of around 1–2.5 km are common^87,88^. Most agricultural lots in our study area are of around 500 m × 800 m and thus their content can be easily assessed from the public roads where nest boxes are installed. At distances greater than 500 m, the land use can rarely be identified from the road and thus we should ask for authorization to each farmer to enter their property to identify the most distant crops. This would impose huge additional logistic constrains to our work as we should arrange visits to probably more than 160 farms (75% of the farms in our study area are smaller than 500 ha^89^). Therefore, we decided to define a 500 m radius territory for each nest box as a measure that has biological meaning and support from the literature and at the same time to allows us to optimize the field work. Using ArcGis program version 9.3^90^, the buffer of 500 m radius surrounding each nest box was generated from the tool imagery base map and then each lot within the buffers was digitalized and transformed into a polygon. Once in the field, the land use corresponding to each polygon (lot) was registered visually. The following land use categories were considered: native forest, exotic groves, pastures (natural or implanted), soybean, stubble (included plowed and fallow fields), sunflowers, corn, cereals (wheat, rye) and, peridomestic zone (areas devoted to human activities such as houses and barns). For statistical analyses we used the surface of the most representative land uses (those that cover at least 10% of the surface of at least one sampling area) within a radius of 500 m around each nest box: native forest, pastures, soybean, corn and stubble. As land uses change seasonally as the breeding season progresses, we registered the different land uses in two stages of the breeding season, at the stage of egg-laying (mid-September–October) and the state of chick-rearing (mid-November–December).

To assess, if weather affected American kestrel breeding parameters, monthly rainfall values (mm) were provided by police stations, a private field in the intensive farmland area and INTA (National Institute of Agricultural Technology). To assign weather variables to the nest boxes, we used the source of information located closest to each nest box. To assess the effect of temperature we used CHELSA climate dataset^91^ (Climatologies at High Resolution for the Earth’s Land Surface Areas, available at http://www.chelsa-climate.org) which includes the monthly mean, minimum and maximum temperature patterns for minimum and maximum temperatures for the study area. Mean temperatures were extracted from Climatic Research Unit time-series (CRU)^92^. To assess, if weather affected breeding parameters, we divided the American kestrel breeding phenology into three stages: prelaying period (winter, June to August), laying period (onset of laying, September to October) and nestling period (chick rearing and fledging, November and December). During these periods we calculated rainfall accumulated, and the average minimum, maximum and mean temperature for each sampling area. For the nestling period, we also used separately the monthly rainfall of November and December. We also calculated rainfall accumulated during the reproductive season (August to December) for each sampling area.

### Breeding phenology

Nest boxes were monitored from 2011 to 2016. During the winter all nest boxes were cleaned and filled with wood shavings ready for the breeding period. Since mid-October, the beginning of the breeding season^45^, all nest boxes were checked every week to assess occupation, laying date and clutch size. Once the clutch was completed, the nest box was visited again just around the presumed hatching date^51^. We considered the estimated incubation period, average of 30 days^47^. During visits to each sampling area, we determined for each nest box the laying date (the date of the first egg), clutch size (number of eggs), productivity and breeding success. The date of the first egg was adjusted to the number of days since September 1st. When we didn’t have the exact date we used back-calculation from hatching dates estimated from direct observation or by calculating it according to the nestlings size and feather development^93^. American kestrels lay one egg every 2 days^47^. When the clutch had already started before our first visit, 2 days were discounted for each egg present in the nest boxes to calculate the laying date. When the clutch was complete at the time of the first visit, the laying date was calculated by subtracting the estimated age of the chicks that day, plus the average 30 days of incubation, plus the number of days necessary to complete the clutch minus one egg (since the incubation period in the American kestrel usually begins when the penultimate egg is laid^47^).

Productivity was defined as the number of fledglings that fledged per pairs attempting breeding and the breeding success as pairs that successfully reared at least one nestling from the total number of pairs that started breeding. We considered a breeding attempt successful when at least one nestling reached 80% of the age necessary to fledge^94^, in this case, approximately 22 days^93^.

### Statistical analyses

Using the software R 3.6.1^95^, generalized linear mixed-effects models (GLMM) and linear mixed-effect models (LMM) were built to examine variation in American kestrel reproductive parameters (occupation, laying date, clutch size, productivity, and breeding success) between the three sampling areas (entered as a factor with three levels, PLNR, TF and IF) for the years 2012 to 2016. We excluded the year 2011 because nest boxes were not installed in the intensive farmland until 2012.

To control for non-independence by the repeated measurements on the same sampling unit (nest box) in successive years, the nest box ID and year were included as random factors. The laying date model was built using LMM with the function lmer from package *lme4*^96^. The clutch size model was built using a Conway-Maxwell-Poisson distribution with a log link function to avoid problems with under-dispersed data. For analyzing productivity, we used a binomial negative error distribution for over-dispersed count data, using the function glmmTBM from package *glmmTBM*^97^. Breeding success and occupation models were built using binomial distribution and logit links functions with the response variable being 1 (successful/occupied) or 0 (not successful/not occupied) using the function glmer, package *lme4*^96^. We also performed a post hoc pairwise comparison between levels of the variables sampling area using the Tukey test to examine the levels in which there were differences in the different response variables.

To evaluate the effect of different land uses and weather (rainfall and temperature) on the same response variables (laying date, clutch size, productivity and breeding success) generalized linear mixed-effects models (GLMM) and linear mixed-effect models (LMM) were built for data from the years 2012 and 2014 to 2016. We excluded the 2011 and 2013 breeding seasons because nest boxes were not installed in the intensive farmland until 2012 and because in 2013 information on land uses was not available. Nest box occupation was not modeled since in agricultural areas occupation was practically 100%, so there was no variability to explain in those areas. In all models we used as covariates the surface of the most representative land uses: native forest, pastures, corn, soybean, and stubble. In all models to control for non-independence the nest box ID was included as a random factor and the term year was not included as a random factor to maintain the simplest random effects structure and avoid convergence issues^98^. Collinearity was evaluated using Pearson correlation. Variables that presented a Pearson correlation r > 0.60 were sequentially eliminated. Multi-collinearity was assessed by calculating generalized variance inflation factors (VIF) using the *usdm* package^99^. Laying date model was built using LMM. For this model, only three years were considered from 2014 to 2016 because in 2012 land uses were assessed only in the chick-rearing period. As explanatory variables, we initially considered rainfall data from the prelaying period (winter, June to August) and laying period (September to October), temperature data from prelaying period (Tmedia_prelaying) and from laying period (Tmin_laying and Tmax_laying), land uses (native forest, pastures, corn, and stubble, soybean was excluded because in laying period, there is no presence of this crop). Due to high collinearity, the variables Tmedia_prelaying and Tmax_laying were excluded from the model. The model was run with the remaining explanatory variables.

The clutch size model was built using a Conway-Maxwell-Poisson distribution with a log link function to avoid problems with under-dispersed data. Again for this model, only three years from 2014 to 2016 were used because in 2012 land uses were assessed only in the chick-rearing period. We used as covariates: laying date, rainfall data from the prelaying period (winter) and laying period, temperature data from the prelaying period (Tmedia_prelaying) and temperature from the laying period (Tmin_laying and Tmax_laying) land uses (native forest, pastures, corn, and stubble; soybean was excluded because in laying period, there is no presence of this crop). Due to high collinearity, the variables Tmedia_prelaying and Tmax_laying were also excluded from the model. The model was run with the remaining explanatory variables.

The productivity model was constructed using a binomial negative error distribution for over-dispersed count data. We used as covariates laying date, rainfall accumulated during the reproductive season, rainfall of the prelaying period (winter), rainfall from November and from December (nestling period), minimum (Tmin_neslting) and maximum (Tmax_nestling) temperatures registered during the nestling period, and land uses (native forest, pastures, corn, soybean and stubble). We excluded from the models the rainfall accumulated during the reproductive season and minimum temperature during the nestling period (Tmin_nestling) for being highly correlated. Finally, breeding success models were built using binomial distribution and logit links functions with the response variable being 1 (successful) or 0 (not successful). We included as covariates, laying date, rainfall accumulated during the reproductive season, rainfall from November and from December (nestling period), minimum temperature from the laying period (Tmin_laying), minimum (Tmin_neslting) and maximum (Tmax_nestling) temperatures registered during the nestling period, and land uses (native forest, pastures, corn, soybean and stubble). Rainfall accumulated during the reproductive season and minimum temperatures (Tmin_nestling) during the nestling period were excluded due to high collinearity.

To simplify the maximal models, each explanatory variable was tested for significance in turn following the backward stepwise procedure, removing sequentially non-significant terms from the full model and retaining only the significant explanatory variables^100^. We considered a variable was significant with p ≤ 0.05. The result was the most adequate model for explaining the variability in the response variable, where only significant explanatory variables were retained.

### Ethics statements

No handling or sampling of birds were done for the specific purpose of this particular study, although we did so for other studies. Fieldwork and all field procedures were conducted under permits from the Subsecretaría de Ecología (La Pampa province, Argentina) to work in Parque Luro Natural Reserve and from the Dirección de Recursos Naturales (La Pampa province, Argentina).

## Supplementary information

Supplementary Information.
