# Supplementary material for: Weather and agricultural intensification determine the breeding performance of a small generalist predator
Source: Sci Rep. 2020 Nov 12;10:19693. doi: 10.1038/s41598-020-76609-x (PMC7665201; doi:10.1038/s41598-020-76609-x)
Supplement: Supplementary file 1 — Supplementary Information. [file 41598_2020_76609_MOESM1_ESM.docx]

**SUPPLEMENTARY MATERIAL**

**Weather and agricultural intensification determine the breeding performance of a small generalist predator**

**Paula M. Orozco-Valor ^1,2*^ & Juan M. Grande^1^**

*^1^Instituto de las Ciencias de la Tierra y Ambientales de La Pampa (INCITAP) -Consejo Nacional de Investigaciones Científicas y Técnicas de Argentina (CONICET), Mendoza 109, (6300) Santa Rosa, La Pampa, Argentina. ^2^Centro para el Estudio y Conservación de las Aves Rapaces en Argentina (CECARA), Facultad de Ciencias Exactas y Naturales, Universidad Nacional de La Pampa, Avenida Uruguay 151, (6300) Santa Rosa La Pampa, Argentina.*

^*^Email address: [pauoro_07@hotmail.com](mailto:pauoro_07@hotmail.com)

**Table S1**. Model analysis GLMM and LMM showing the effect of different explanatory variables over laying date, clutch size, productivity and breeding success of American kestrel.

| Model | Explanatory variable | Estimate | SE | X^2^ | df | P |
| --- | --- | --- | --- | --- | --- | --- |
| Laying date | intercept | 52.071 | 4.963 |  |  |  |
|  | forest | 0.169 | 0.045 | 14.508 | 1 | <0.001 |
|  | tmin laying | -1.211 | 0.565 | 4.666 | 1 | 0.031 |
|  | pasture |  |  | 0.328 | 1 | 0.566 |
|  | corn |  |  | 0.154 | 1 | 0.694 |
|  | stubble |  |  | 0.109 | 1 | 0.741 |
|  | prelaying rain |  |  | 0.793 | 1 | 0.373 |
|  | laying rain |  |  | 1.983 | 1 | 0.159 |
| Clutch size | intercept | 1.168 | 0.048 |  |  |  |
|  | laying date | -0.004 | 0.001 | 14.065 | 1 | <0.001 |
|  | forest |  |  | 2.356 | 1 | 0.124 |
|  | pasture |  |  | 1.019 | 1 | 0.312 |
|  | corn |  |  | 0.226 | 1 | 0.634 |
|  | stubble |  |  | 0.0003 | 1 | 0.986 |
|  | prelaying rain |  |  | 0.021 | 1 | 0.883 |
|  | laying rain |  |  | 0.777 | 1 | 0.377 |
|  | tmin laying |  |  | 0.049 | 1 | 0.823 |
| productivity | intercept | 6.156 | 1.958 |  |  |  |
|  | November rain | -0.003 | 0.001 | 11.342 | 1 | <0.001 |
|  | Tmax_nestling | -0.171 | 0.069 | 6.014 | 1 | 0.014 |
|  | laydate | -0.004 | 0.001 | 3.606 | 1 | 0.057 |
|  | forest |  |  | 0.244 | 1 | 0.621 |
|  | pasture |  |  | 0.399 | 1 | 0.527 |
|  | corn |  |  | 0.130 | 1 | 0.717 |
|  | stubble |  |  | 0.493 | 1 | 0.482 |
|  | soybean |  |  | 0.484 | 1 | 0.486 |
|  | December rain |  |  | 0.193 | 1 | 0.660 |
|  | prelaying rain |  |  | 0.169 | 1 | 0.680 |
| Breeding success | intercept | 18.762 | 8.291 |  |  |  |
|  | Pasture | 0.017 | 0.007 | 5.056 | 1 | 0.024 |
|  | November rain | -0.010 | 0.003 | 7.436 | 1 | 0.006 |
|  | Tmax_nestling | -0.061 | 0.029 | 4.376 | 1 | 0.036 |
|  | laydate |  |  | 0.343 | 1 | 0.558 |
|  | Forest |  |  | 0.001 | 1 | 0.974 |
|  | Corn |  |  | 0.121 | 1 | 0.728 |
|  | Stubble |  |  | 1.582 | 1 | 0.208 |
|  | Soybean |  |  | 0.612 | 1 | 0.433 |
|  | December rain |  |  | 0.267 | 1 | 0.604 |
|  | prelaying rain |  |  | 2.683 | 1 | 0.101 |
|  | Tmin_laying |  |  | 0.040 | 1 | 0.840 |
